# Wound Technic

**Published:** 1916-05

**Authors:** James A. Macleod

**Affiliations:** Buffalo, N. Y.


### BUFFALO MEDICAL JOURNAL

Yearly Volume 71 MAY, 1916 No. 10

### ORIGINAL ARTICLES

**The right is reserved to decline papers not dealing with practical medical and surgical subjects, and such as might offend or fail to interest readers. Contributors are solely responsible for opinions, methods of expression and revision of proof.**

### Wound Technic

By JAMES A. MacLEOD, M. D., M. R. C. S., Eng., F. A. C. S.,

Buffalo, N. Y.

Our Science, or I may call it our Art, has advanced during the last few years in an incredible manner; operative technic is becoming nearer perfection every year; the sterilization of our equipment has become reasonably certain; but the skin is no nearer perfect sterilization today than it was years ago. Opening on the surface of the skin are myriad tubules leading up from the sweat and sebaceous glands situated in the deeper portions of the skin; these are inhabited by countless numbers of bacteria, the kind depending largely upon the personal cleanliness of the patient, but of which the staphylococcus albus is usually the predominant organism. It is true that the staphylococcus albus is not a very virulent organism, but it is able to cause suppuration in the wound and give rise to trouble which may be far reaching in its importance. Besides the staphylococcus albus we have other organisms usually present, and the part played by them depends upon their kind and number. It is quite evident, from the deep and wide distribution of the bacteria, that it is practically impossible to completely sterilize the skin without destroying the skin itself. Following a careful pre-operative preparation of the skin, cultures taken are negative; but taken at the completion of an operation, lasting more than a few minutes, very often show bacterial growth; this may be explained by the fact that the manipulations of the operation and the heat present are favorable to the migration of bacteria from the deeper portions of the tubules to the surface. The skin, notwithstanding elaborate precautions taken in its preparation, is, therefore, liable to become infective soon after the commencement of the operation; how soon it becomes so depends largely on how much of the chemical used in its preparation is left on the surface, and how long it remains there. Various chemical and organic substances have been used, and are being used; but iodine is probably the one substance par excellence at the present time; most men, however, recognizing the danger of irritating the delicate peritoneal covering of the abdominal viscera that may come in contact with the skin, remove it by alcohol; but by so doing, the time of the skin remaining sterile is much shortened, as the presence of the iodine is a direct hindrance to the existence of living bacteria.

In abdominal work the presence of chemical irritants on the surface and the re-appearance of bacteria are of great importance, and I shall discuss the problem, first, in regard to the abdominal viscera, and second, in regard to the wound:

**First**: Even where the iodine is followed by alcohol, it may be conceded that traces of it are left; any iodine left would act as a direct irritant to the delicate peritoneal coat of the viscera brought out on the surface of the skin. Later on in the course of the operation bacteria may make their re-appearance; the time of such occurrence depends on the amount of iodine left on the surface, the amount of traumatism to the wound and the amount of heat present. Bacteria, being present, are liable to cause an infection of the peritoneal coat of the viscus or viscera resting on the surface of the skin; the degree of infection depends on the kind of bacteria present and the number, and may vary from a mild one to a severe and wide-spread inflammation. Probably we have a mixture of both these irritations in the majority of cases. The viscus or viscera are returned to the abdomen and there undergo an inflammatory reaction, depending on the amount of the irritation or infection received on the surface of the skin. Where the reaction is due to chemical irritation it may be of a mild character, but yet sufficient to cause the gluing of viscera together and the formation of adhesions. Where the reaction is due to bacterial infection, it is usually of a low grade type and it is readily taken care of by the peritoneum; it is, however, sufficient to cause the gluing of viscera together, the formation of adhesions and a partial paresis of the intestinal muscles, with all of its distressing signs and symptoms.

**Second**: The re-appearance of bacteria on the surface of the skin is especially important in the question of wound infection, which depends upon a number of factors: 1: The presence of bacteria, their kind and number. 2: The presence of suitable culture material in the shape of blood or blood clot. 3: The amount of traumatism of the tissues of the wound; the fat is the most fragile of the layers, least resistant to traumatism and it is, therefore, the commonest seat of infection of all the layers. 4: The sewing of the skin; here the needle perforates the skin and must necessarily pass through numerous ducts containing bacteria, which are carried by the needle in its passage. When an infection does occur it may vary in intensity from a mild irritation to a wide-spread inflammation; furthermore it may have remote results in the shape of a septic infarction of the lung, a condition very commonly looked upon as a post-operative pneumonia due to the anaesthetic, and other remote secondary infections. It is true that wounds do heal by first intention even in spite of the presence of bacteria, but such depends upon, first, the high resisting power of the tissues, second, minimized traumatism of the tissues of the wound, and third, the low degree of virulence of the bacteria present.

Following the operation we may have immediate trouble with the wound in the shape of complete rupture, due to the premature disappearance of the suture material, or later in the shape of a ventral hernia. In the former the calamity is explained by Oschner as due to a chemical action taking place by the presence of some enzyme, causing a softening and solution of the suture material; this may be the explanation but I am inclined to think that faulty preparation of the gut plays a considerable part.

From the above considerations it is plainly seen that the exclusion of the skin from the actual field of operation, as so ably pointed out by Moynihan and others, is, or should be, an extremely important part of our operative technic. The technic used by the surgeons who recognize the importance of skin exclusion vary somewhat, but the basic principles are the same. That which I have been using for more than a year may be outlined as follows: **First**: The skin is shaved carefully, scrubbed thoroughly with soap and water, using a soft wash cloth and plenty of water, and then rubbed with alcohol; this is done to insure a clean dry surface free from oil or fat; when practicable a steam or cabinet bath should be given the day preceding the operation. **Second**: The field is covered by a dry sterile dressing or towel; wet antiseptic dressings should be avoided to prevent any possible maceration of the superficial epithelium, which is favorable to the growth of bacteria; lately I have had no dressings applied at all. **Third**: After the patient is anaesthetized the skin of the field of the operation is painted with a 5% alcoholic solution of iodine, which is allowed to dry; this is not washed off by alcohol but allowed to remain with the idea of its acting on any bacteria that might migrate to the surface from the deep tubules. **Fourth:** The operative field is surrounded in the usual manner by sterile towels and sheets. **Fifth:** The skin incision is made of ample length for free work and any bleeding stopped by the application of hemostats. **Sixth:** Cloths are clamped to the edges of the incision, taking in as much fat as possible and covering the hemostats; the cloth clamps are preferably placed on the deep sides of the cloths; the cloths are clamped together at the extremities of the incision and the skin is then found to be completely excluded from the further steps of the operation: I find that the domestic bathroom wash cloths answer the purpose exceedingly well. **Seventh:** The abdomen is opened through the various layers to the extent of the incision. **Eighth:** During the balance of the operation exposure of the viscera, omentum, intestines, etc., not intimately engaged in the operation, is prevented by the application of large abdominal packs wrung out of hot normal saline solution; if the operation be in any way prolonged the packs should be changed from time to time to insure that the parts are not only kept covered and free from traumatism, but are also kept warm; it is often noted that the intestines, in close proximity to the focus to be operated upon, are at the commencement of the operation in an acutely congested condition, and that, at the termination of the operation, they have regained their normal color. **Ninth:** The abdominal work being completed the viscera are returned to their normal position, special care being taken with the replacement of the omentum, and the peritoneum closed; in regards to the omentum I may report that within the last month I have operated upon three patients, who were suffering from colonic stasis due to post-operative adhesions; in each case the omentum was found bunched and adherent to the ascending colon and adjacent abdominal wall, and causing obstruction. **Tenth:** Taking into consideration the possible risk of the premature disappearance of suture material I have instituted at this stage of the operation a row of interrupted kangaroo stitches through the muscles and aponeurosis; the aponeurosis or rectal sheath is approximated by a continuous chronic gut stitch and the kangaroo stitches are tied. **Eleventh:** The skin cloths are carefully removed from the field of operation and discarded. **Twelfth:** The skin is again painted with the iodine solution. **Thirteenth:** The wound is swabbed out by a cloth wet with hot saline solution and any bleeding points controlled by ligature. **Fourteenth:** 3 or 4 interrupted silkworm stitches are inserted from within outwards and embracing all layers with the exception of the peritoneum (but are not tied); these are inserted from within outwards to preclude the carrying of germs down from the surface and tubules to the deeper parts of the wound. **Fifteenth:** The edges of the incision are approximated by a row of metal clips, which do not perforate the skin; the reason for the use of approximating clips instead of a stitch is again to obviate the chance of carrying bacteria into the deeper tissues. **Sixteenth:** The silkworm stitches are either carried through sections of tubing and tied, or tied over a metal frame; by so doing the tension exerted is kept even and the skin is not cut by the stitches in their pressure downwards. **Seventeenth:** The operation is completed by painting again the skin field with the iodine solution. **Eighteenth:** The dressings are applied dry and kept in position by a wide strip of adhesive plaster, reaching nearly to the spine on each side, and from the ribs to the ilium; no bandage or binder is applied; the patient finds this mode of dressing very comfortable as the abdominal muscles are evenly supported, and I find that the patient suffers less from distension and its accompanying distress.

The after-care: The adhesive binder is split down the middle on the second or third day; the wound is inspected, again painted by the iodine solution and dry dressings applied. The binder is brought together over the dressings by means of a running string placed through perforations on the opposite sides. On the seventh day the clips are removed and on the tenth the silkworm stitches are removed.

The procedure as outlined above is elaborate and increases the time of the operation, but the benefits accruing are well worth the extra time and trouble. To summarize the benefits I may say that they are as follows: First: The use of skin cloths, the frequent painting of the surface of the skin with the 5% alcoholic solution of iodine, the insertion of the silkworm stitches from within outwards and the approximation of the skin by metal clips, which do not perforate the skin, practically obviates wound infection.

Second: The absence of wound infection minimizes the danger of septic pulmonary infarction and other remote secondary infections.

Third: The protection of the abdominal viscera from chemical irritation or bacterial infection by the use of wound cloths and their protection from traumatism by the use of hot abdominal packs minimizes the formation of post-operative adhesions, post-operative distension, etc.

Fourth: The use of the row of interrupted kangeroo stitches obviates the danger of immediate rupture of the wound by the premature disappearance of the suture material, and minimizes the danger of the formation of a ventral hernia later on.

448 Delaware Avenue.

